# Targeted Sequencing of Ascites and Peritoneal Washing Fluid of Patients With Gastrointestinal Cancers and Their Clinical Applications and Limitations

**DOI:** 10.3389/fonc.2021.712754

**Published:** 2021-07-15

**Authors:** Go Eun Bae, Seok-Hwan Kim, Min Kyung Choi, Jin-Man Kim, Min-Kyung Yeo

**Affiliations:** ^1^ Department of Pathology, Chungnam National University School of Medicine, Daejeon, South Korea; ^2^ Department of Surgery, Chungnam National University School of Medicine, Daejeon, South Korea

**Keywords:** gastrointestinal, cancer, ascites, peritoneal washing, cytology, liquid biopsy

## Abstract

Cytology from gastrointestinal (GI) cancers is frequently obtained from ascites and peritoneal washing fluids. Examination of ascites and peritoneal washing fluids from patients with GI cancers can help in the tumor staging and prognosis. Tumor-derived DNA in these cytology samples can be a target for next generation sequencing (NGS). Targeted NGS was evaluated in ascites and peritoneal washing samples obtained from 33 patients with GI cancers. These sequences were compared with those from tumor tissue samples, and correlated with cytopathologic findings of the ascites and peritoneal fluid samples. The correlation between fluid and tissue genotyping results was 25%, with a sensitivity of 21.43%. The volume of tumor contained within the fluid samples was low, ranging from ~0 to 10%. Importantly, the sensitivity of detection of somatic mutations in the fluid samples could be increased to 69.2% by assessing samples containing >2% tumor volume. Evaluation of cells from ascitic fluid showed the presence of KRAS, TP53, and CDH1 mutations in 33, 13, and 7%, respectively, of patients with pancreatic cancer, and the presence of KRAS, TP53, and APC mutations in 25, 12, and 13%, respectively, of patients with gastric cancer. Ascites of one of the latter patients acquired KRAS mutation, which was a novel mutation during metastasis. Targeted NGS of ascites and peritoneal washing fluid have clinical implications, as well as limitations, in patients with GI cancers. NGS-based cytology examination may expand cytomolecular practices in GI cancer patients.

## Introduction

Cytology specimens for diagnostic purposes can be obtained by exfoliation or aspiration of cells from cancer patients. Ascites and peritoneal washing fluids are common sources of cell specimens from gastrointestinal (GI) cancer patients (1, 2). Ascites, defined as the abnormal collection of fluid in the peritoneal cavity, can occur due to cancers and other diseases, including liver cirrhosis, heart failure, chronic renal failure, and peritoneal infection. Cytologic evaluation of ascitic fluid can identify the presence of malignant cells and rule out benign causes. Peritoneal washing fluid is obtained by irrigation of the peritoneal cavity with normal saline solution. Cytologic evaluation of peritoneal washing fluid can detect occult cancer cells in GI cancer patients, even without the collection of ascitic fluid. Peritoneal metastasis is the most common pattern of recurrence and cause of death in patients with cancers of the GI tract, making cytologic evaluation of peritoneal washing fluid important in determining cancer stage and predicting recurrence in these patients.

Cell samples can be easily obtained and repetitively sampled from cancer patients. Drawbacks of cytologic examination of fluid samples include their low cellularity, the heterogeneity of their cell populations, and the low relative volume of tumor contained within these samples. Samples from the peritoneal cavity frequently contain large numbers of background reactive mesothelial and inflammatory cells, making it difficult to detect relatively small numbers of tumor cells. The ability of peritoneal cytology to diagnose and classify cancers is relatively low, with diagnostic sensitivity ranging from 50 to 70% (3). Methods developed to improve the diagnostic performance and overcome the low sensitivity of these cytologic methods include assays of specific cancer protein markers in these samples (4, 5).

Ascites and peritoneal fluid samples obtained from patients with malignancies are enriched in tumor proteins as well as tumor DNA. Reliable genetic approaches using tumor-derived DNA can be used as ancillary tests for cytological diagnosis. Next generation sequencing (NGS) can analyze mutations in DNA and identify pathogenic variants with low allelic fraction (AF). NGS of liquid biopsy samples, such as blood, urine, effusion fluids, and cerebrospinal fluid, has shown clinical application in cancer patients (6). NGS of cell samples has also shown clinical utility in diagnosis, including the evaluation of tumor heterogeneity, emergence of drug resistance, and determination of minimal residual disease in cancer patients (7). Cytology specimens differ in their relative amount and type of tumor-derived DNA. NGS evaluation of cells extracted from ascites and peritoneal washing fluid can enhance diagnostic pathology.

This study hypothesized that NGS-based testing can be utilized to improve the diagnostic of patients with GI cancers by studying cell samples isolated from ascites and peritoneal washing fluids of these patients. Targeted NGS of these ascites and peritoneal washing samples was performed to explore the genomic features of patients with malignant, suspicious, and benign cytology. Genetic variants found in these cytology specimens were compared with those of tumor tissues and correlated with clinicopathologic features. Finally, this study assessed the performance of targeted NGS in cells extracted from ascites and in peritoneal washing samples from patients with GI cancers.

## Materials and Methods

### Patients and Sample Collection

This retrospectively study was approved by the Chungnam National University Hospital institutional review board (IRB file no. CNUH 2020-09-015), which waived the requirement for informed consent. All samples were provided by the Biobank of Chungnam National University Hospital, a member of the Korea Biobank Network.

Ascites and peritoneal washing samples obtained from 33 patients from January 2018 to December 2020 were analyzed by the pathology laboratory at Chungnam National University Hospital (Daejeon, Korea). These 33 patients consisted of 27 patients with cancers, including thirteen with pancreatic cancer and fourteen with gastric cancer, and six with non-malignant (benign) diseases, such as renal failure and peritonitis. Ascitic fluid was obtained from 13 patients with pancreatic cancer, eight with gastric cancer, and six with non-malignant diseases; whereas, peritoneal washing fluid samples were obtained from six patients with gastric cancer. Paired tumor samples were also obtained from 10 cancer patients, including two with pancreatic and eight with gastric cancers; these samples were fixed in formalin and embedded in paraffin blocks.

**Figure 1:**
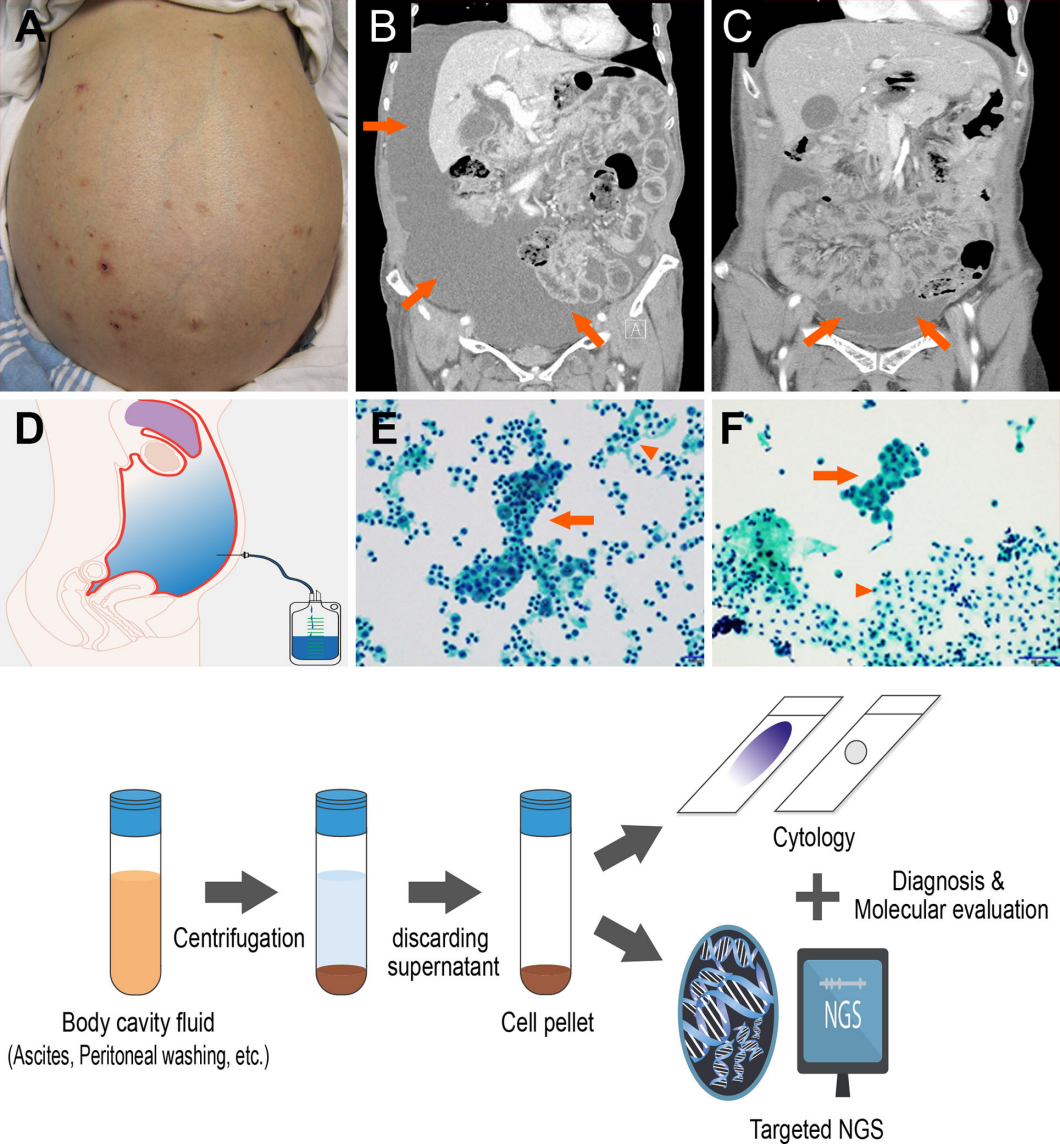
Ascites and peritoneal washing cytology from the gastrointestinal cancer patients **(A)** Patients with swollen abdomen with massive ascites. Abdominal computed tomography scans showing **(B)** a large amount of ascites, **(C)** mild ascites irrigated with peritoneal washing. **(D)** Cytology samples can be collected from paracentesis. **(E)** Ascites and **(F)** peritoneal washing cytology contains tumor cells (arrow) with abundant background mesothelial cells and inflammation cells (arrow head). (Below) Cytology sample processing to diagnosis and targeted sequencing.

Ascites and peritoneal washing samples were submitted to the pathology department and processed within 12 h of collection according to standard protocols (**Figure 1**). Briefly, samples were centrifuged at 1,200*g* for 5 min, and each cell pellet was transferred to liquid-based solution vials to make liquid-based (ThinPrep) cytology slides. The slides were examined by two pathologists (MKY and GEB), with a diagnosis of malignant, suspicious for malignancy, atypia, benign cellular changes, or negative for malignancy reached by consensus. The tumor volume in the slides were calculated by a total area of tumor cells (cm^3^)/total area of cells in each slide (cm^3^) considering their 3D clusters. Each cell volume was measured by width × width × length × 0.52. The remainder of each cell pellet was decanted into a separate conical tube and stored immediately at −80°C for subsequent DNA extraction and targeted NGS.

### Sample Preparation, Library Preparation, and Sequencing

Cell-derived DNA was extracted from each 300 μl cell pellet using DNeasy Blood & Tissue Kits (Qiagen, Hilden, Germany) and from formalin-fixed, paraffin-embedded (FFPE) tissue samples using QIAamp DNA FFPE Tissue Kits (Qiagen), according to the manufacturer’s instructions. The extracted DNAs were quantified using a Qubit 3.0 fluorometer (Thermo Fisher Scientific, Waltham, MA, USA) and a 4150 TapeStation System (Agilent, Santa Clara, CA, USA).

Libraries were constructed from 10 ng aliquots of cell pellet and FFPE tissue-derived DNA samples using a customized cancer panel, which allows the detection of mutations in 58 cancer-related genes (**Table 1**), which were selected using open data base (TCGA (https://tcga-data.nci.nih.gov), COSMIC (https://cancer.sanger.ac.uk/cosmic), Intogen (https://www.intogen.org/) and showed >1% hotspot mutation. Cell pellet-derived DNA samples were sequenced to a median depth ×5,000 and tissue-derived DNA samples to a median depth ×1,000, revealing a broad range of genetic alterations. Libraries were prepared using Ion Ampliseq Library Kits 2.0 (Thermo Fisher Scientific) according to the manufacturer’s instructions. The Ion Express Barcode Adaptors Kit (Thermo Fisher Scientific) was used for sample multiplexing, and libraries were purified using an Agencourt AMPure XP reagent (Beckman Coulter, Danvers, MA, USA). The libraries were quantified using the Qubit 3.0 fluorometer and the 4150 TapeStation System. Templates for the libraries were prepared using the Ion Chef Instrument (Thermo Fisher Scientific) with the Ion 540 Chef Kit (Thermo Fisher Scientific). Multiplexed templates were subjected to sequencing on the Ion S5 XL system (Thermo Fisher Scientific).

**Table 1:** Cancer genes for targeted sequencing.

ACVR2	ADH1B	ALB	APC	APOB	ARHGAP35	ARID1A	ARID1B	ARID2	ATM
ATRX	AXIN1	BRD7	CDH1	CDKN1A	COL11A1	CREBB	CTNNB1	CYP2E1	EGFR
ERBB2	EYS	FAT4	FBN2	FLT3	G6PC	HNF1A	HNRNPA2B1	IHD2	JAK1
JAK2	KEAP1	KIT	KMT2C	KMT2D	KRAS	LRP1B	MAP2K3	NCOR1	NFE2L2
PBRM1	PCLO	PDGRFA	PIK3CA	PREX2	PTEN	RB1	RPS6KA3	RYR2	SETD2
SF3B1	SMAD4	SRCAP	TBL1XR1	TP53	TSC1	TSC2	XIRP2		

### Sequencing Data Analysis

Data were analyzed using Torrent Suite software (5.8.0). Sequencing coverage was analyzed using coverage Analysis (5.8.0.1) plugins, and VCF files were generated using the variant Caller (5.8.0.19) plugins. Variations were annotated using Ion Reporter (5.10.2.0) software. SNVs were defined as having 1) a minimum number of total coverages ≥500, 2) Phred-scaled minimum average evidence per read ≥10, and 3) a minimum variant allele frequency (VAF) ≥1%. The lower limits of detection of SNVs and indels were defined as a 5% allele frequency for 200× coverage and a 2% allele frequency for 500× coverage. Variants with minor allele frequencies ≥1% in large population databases (single-nucleotide polymorphisms) were removed. Select gene rearrangements were detected using an internally developed algorithm. For copy number analysis, a read depth-based method was implemented to report focal, high-level amplifications in regions with mean z-scores ≥12 when compared with a set of normal samples.

Genetic alterations were classified as pathogenic based on publicly available resources and the primary literature. Genetic variants identified were interpreted and categorized as pathogenic, likely pathogenic, variant of uncertain significance or conflicting interpretations of pathogenicity, presumed benign, or benign by their clinical significance in accordance with ClinVar-indexed variants (National Center for Biotechnology Information (NCBI), Bethesda, MD, USA) (8). When assessing mutation frequencies of individual genes, pathogenic, likely pathogenic, and of uncertain significant were defined as mutations, whereas presumed benign and benign variants were excluded. The sequencing data of the 33 cytology and 10 FFPE samples were provided as **Supplementary File 1**.

### Statistical Analysis

Receiver operating characteristic (ROC) curves were constructed to determine the relationship between tumor volume and NGS detection of mutation. The sensitivity, specificity, positive predictive value (PPV), and negative predictive value (NPV) of cytologic detection of mutation in NGS analysis were calculated in reference to the results of tissue samples. All statistical analyses were performed using SPSS version 26.0 for Windows (SPSS, Inc., Chicago, IL, USA) and MedCalc version 19.2.0 for Windows (MedCalc Software, Ltd., Ostend, Belgium).

## Results

### Characteristics of Patients and Cytological Diagnoses

The baseline demographic and clinical characteristics of the 33 evaluated patients are shown in **Table 2**. Ascites samples were obtained from 13 patients with pancreatic cancer, eight with gastric cancer, and six with non-malignant diseases, and peritoneal washing samples were obtained from six patients with gastric cancer. These 33 patients included 20 men and 13 women, ranging in age from 39 to 90 years. The time interval between tissue samples and cytologic specimens ranged from 0 to 42 months. Pancreatic and gastric cancer patients with ascitic fluid showed clinically poorer outcomes, whereas gastric cancer patients with peritoneal washing fluid showed stable disease outcome, without recurrence during follow-up.

**Table 2:** Detailed description of the patients (n = 33).

ID	Sex	Age	Clinical diagnosis	Stage	Time of sampling	Interval*(Months)	Cytology sample	Cytologic diagnosis	Prognosis	Death
Group 1 Pancreatic cancer; assay of ascites (n = 13)										
PC1	69	F	Pancreatic ductal adenocarcinoma	pT2N0Mx	After surgery	10	Ascites	Atypia	Progression	Yes
PC2	46	M	Pancreatic ductal adenocarcinoma	pT3NoMx	After surgery	20	Ascites	Atypia	Progression	No
PC3	66	M	Pancreatic ductal adenocarcinoma	cT3NxMx	At biopsy, no surgery performed	1	Ascites	Atypia	Progression	Yes
PC4	61	F	Pancreatic ductal adenocarcinoma	cT2N1M1	After surgery	17	Ascites	Atypia	Progression	No
PC5	88	M	Pancreatic ductal adenocarcinoma	cT2N1Mx	At biopsy, no surgery performed	0	Ascites	Suspicious for malignancy	Progression	No
PC6	69	M	Pancreatic ductal adenocarcinoma	pT4N0Mx	At biopsy, no surgery performed	18	Ascites	Suspicious for malignancy	Progression	No
PC7	71	M	Pancreatic ductal adenocarcinoma	cT4NxMx	At biopsy, no surgery performed	0	Ascites	Suspicious for malignancy	Progression	Yes
PC8	70	M	Pancreatic ductal adenocarcinoma	cT3N2M1	At clinical diagnosis, no biopsy performed	0	Ascites	Malignancy	Progression	Yes
PC9	90	F	Pancreatic ductal adenocarcinoma	cT3NxM1	At biopsy, no surgery performed	0	Ascites	Malignancy	Progression	Yes
PC10	74	F	Pancreatic ductal adenocarcinoma	cT2N2M1	At clinical diagnosis, no biopsy performed	0	Ascites	Malignancy	Progression	Yes
PC11	69	M	Pancreatic ductal adenocarcinoma	cT3N2M1	At clinical diagnosis, no biopsy performed	6	Ascites	Malignancy	Progression	No
PC12	73	M	Pancreatic ductal adenocarcinoma	pT2N1Mx	After surgery	2	Ascites	Malignancy	Progression	Yes
PC13	61	M	Pancreatic ductal adenocarcinoma	cT2NxM1	At clinical diagnosis, no biopsy performed	0	Ascites	Malignancy	Progression	No
Group 2 Gastric cancer, assay of ascites (n = 8)										
GC1	64	F	Gastric poorly cohesive carcinoma	cT4N(+)Mx	At biopsy, no surgery performed	5	Ascites	Malignancy	Progression	No
GC2	79	F	Gastric poorly cohesive carcinoma	pT4N3Mx	After surgery	10	Ascites	Malignancy	Progression	Yes
GC3	49	F	Gastric poorly cohesive carcinoma	pT3N0Mx	After surgery	42	Ascites	Malignancy	Progression	Yes
GC4	73	F	Gastric poorly cohesive carcinoma	pT4N3Mx	At biopsy, no surgery performed	36	Ascites	Malignancy	Progression	Yes
GC5	58	M	Gastric poorly cohesive carcinoma	cT4N0M1	After surgery	4	Ascites	Malignancy	Progression	No
GC6	53	M	Gastric poorly cohesive carcinoma	cT4N(+)Mx	At biopsy, no surgery performed	0	Ascites	Malignancy	Progression	No
GC7	48	M	Gastric poorly cohesive carcinoma	cT4N(+)Mx	At biopsy, no surgery performed	5	Ascites	Malignancy	Progression	Yes
GC8	39	F	Gastric tubular adenocarcinoma	cT4N(+)Mx	At biopsy, no surgery performed	0	Ascites	Malignancy	Progression	NO
Group 3 Gastric cancer, assay of peritoneal washing (n = 6)										
GC_W1	86	F	Gastric tubular adenocarcinoma	pT4N3Mx	After surgery	0	Peritoneal Washing	Atypia	No recur	No
GC_W2	83	F	Gastric tubular adenocarcinoma	pT3N2Mx	After surgery	0	Peritoneal Washing	Suspicious for malignancy	No recur	No
GC_W3	59	M	Gastric tubular adenocarcinoma	pT4N3Mx	After surgery	0	Peritoneal Washing	Malignancy	No recur	No
GC_W4	63	M	Gastric tubular adenocarcinoma	pT1N0Mx	After surgery	0	Peritoneal Washing	Negative for malignancy	No recur	No
GC_W5	82	F	Gastric tubular adenocarcinoma	pT1N0Mx	After surgery	0	Peritoneal Washing	Negative for malignancy	No recur	No
GC_W6	69	M	Gastric tubular adenocarcinoma	pT1N0Mx	After surgery	0	Peritoneal Washing	Negative for malignancy	No recur	No
Group 4 Control, benign disease, assay of ascites (n = 6)										
MES1	40	M	Chronic renal failure	NA	At biopsy, no surgery performed	0	Ascites	NA	Not applicable	No
MES2	56	M	Acute renal failure	NA	At biopsy, no surgery performed	0	Ascites	NA	Not applicable	No
MES3	52	F	Peritonitis	NA	At biopsy, no surgery performed	0	Ascites	NA	Not applicable	No
MES4	82	M	Chronic renal failure	NA	At biopsy, no surgery performed	0	Ascites	NA	Not applicable	No
MES5	69	M	Peritonitis	NA	At biopsy, no surgery performed	0	Ascites	NA	Not applicable	No
MES6	60	M	Chronic renal failure	NA	At biopsy, no surgery performed	0	Ascites	NA	Not applicable	No

*Interval between biopsy and cytology (Months); NA, Not applicable.

### Comparison Analysis of Cytology and Tissue Samples

**Figure 2:**
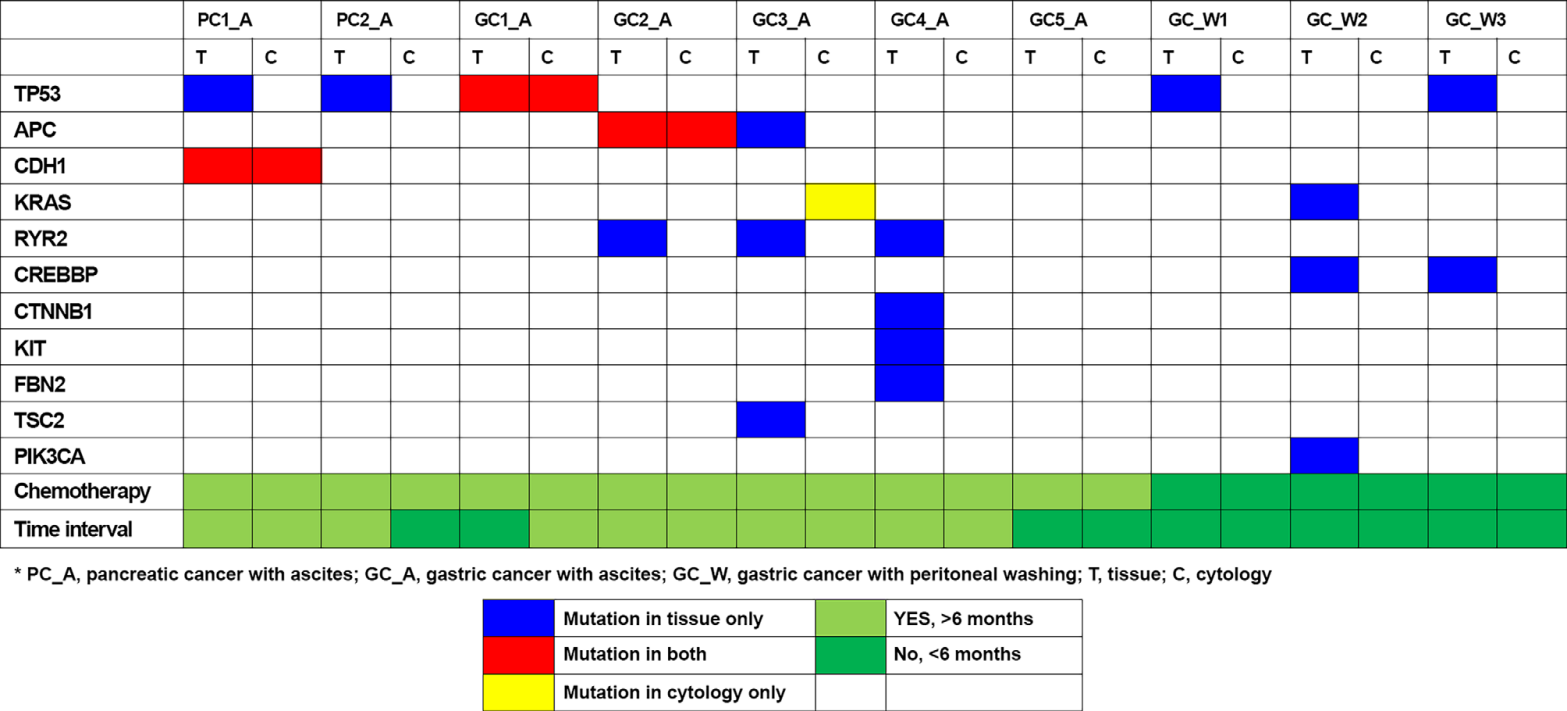
Genomic mutation detection using ascites and peritoneal washing cytology and comparison to tissue sample results.

Genetic variants, as determined by NGS evaluation, were compared in the ascites/peritoneal washing samples and the FFPE tissue samples from 10 patients who underwent surgery or biopsy (**Table 3**). Ascites samples were evaluated in two patients with pancreatic cancer and five with gastric cancer, and peritoneal washing samples were evaluated in three patients with gastric cancer (**Figure 2**). Three patients were found to have the same mutation. Tissue samples from both patients with pancreatic cancer had CDH1 and TP53 mutations, with the same CDH1 mutation also detected in the ascites samples. Tissue samples from gastric cancer patients were found to have TP53, APC, RYR2, CTNNB1, KIT, FBN2, TSC2, and PIK3CA mutations, with TP53 and APC mutations also detected in the paired ascites samples. KRAS mutations were detected only in the ascites samples. Tissue samples from pancreatic cancer patients had TP53, KRAS, CREBBP, and PIK3CA mutations. In one patient with pancreatic cancer (PC1), targeted genes with high mutational frequency in the primary tumor samples also showed high mutational frequency in the cytology specimens, with a pathogenic CDH mutation having an allele frequency (AF) of 4.6% in the cytology specimen and 54.1% in the paired tissue sample. No mutations were identified in the peritoneal washing samples. The overall concordance rate of somatic variants was 25% (sensitivity = 21.43%, specificity = 50.00%; **Supplementary Table 2**).

**Table 3:** Comparison of NGS results in cytology and tissue samples (n = 10).

Patient	Stage	Cytology sample	Cytologic diagnosis	Tumor volume in cytology samples	NGS results of cytology samples	NGS results of tissue FFPE samples
PC1	pT2N0Mx	Ascites	Atypia	0.1%	CDH1 E880K (4.9%)	CDH1 E880K (54.1%), TP53 C176F (15.5%)
PC2	pT3NoMx	Ascites	Atypia	0.2%	Wild	TP53 R175H (1%), TP53 c.673 2A>G (9.2%)
GC1	cT4N(+)Mx	Ascites	Malignancy	5%	TP53 E339K (52.2%)	TP53 E339K (50.8%)
GC2	pT4N3Mx	Ascites	Malignancy	7%	APC G253S (9.6%)	APC G253S (48.9%), RYR2 R2198H (1%)
GC3	pT3N0Mx	Ascites	Malignancy	7%	KRAS G12R (3.4%)	TSC2 S315L (1.1%), APC R99W (1%), RYR2 D4808N (1%)
GC4	pT4N3Mx	Ascites	Malignancy	8%	Wild	CTNNB1 W66 (1%), KIT D496N (1.1%), FBN2 G264D (1.4%), RYR2 R2198H (1.1%)
GC5	cT4N0M1	Ascites	Malignancy	4%	Wild	Wild
GC_W1	pT4N3Mx	Peritoneal washing	Atypia	0.1%	Wild	TP53 R175H (1.1%)
GC_W2	pT3N2Mx	Peritoneal washing	Suspicious for malignancy	0.5%	Wild	KRAS G13D (16%), CREBBP L551I (51.8%), PIK3CA E542K (29%)
GC_W3	pT4N3Mx	Peritoneal washing	Malignancy	1%	Wild	TP53 E271K (34.3%), CREBBP L551I (41.4%)

### NGS Results of the Cytology

**Figure 3:**
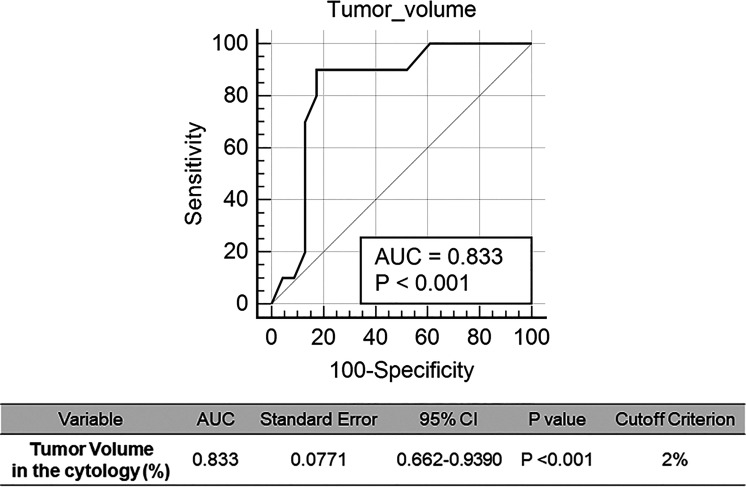
Receiver operating characteristic (ROC) curves for detection of genomic mutation in the cytology sample. Cut-off of the tumor volume in the cytology was calculated.

NGS of cell samples obtained from ascites/peritoneal washing samples is shown in **Table 4**. Evaluation of cells obtained from the ascitic fluid of 13 patients with pancreatic cancer showed atypia in four patients, suspicious for malignancy in three, and malignancy in six. The percentage of tumor cells in the cytology slides ranged from 0.1 to 10%. Evaluation of cells obtained from the ascites of eight patients with gastric cancer showed malignancy in all of them. The percentage of the tumor cells in the cytology slides ranged from 5 to 10%. Cytology evaluation of cells obtained from peritoneal washing samples of six patients with gastric cancer showed atypia in one patient, suspicious for malignancy in one, malignancy in one, and negative for malignancy in three patients. The percentage of the tumor cells in the cytology slides varied from 0 to 1%. As control samples, ascitic fluid was obtained from six patients with non-malignant disease, and all of them harbored benign variants and considered as wild type. NGS evaluation of peritoneal washing fluid from the six patients with gastric cancer and the ascites samples from the six patients with non-malignant disease showed no mutations. Overall tumor volume ranged from ~0–10%. To determine the tumor volume needed to obtain NGS results, a cutoff to determine minimum tumor volume was determined by generating ROC curves. The optimal cutoff for the minimum tumor volume to detect somatic mutations in cytology specimens from cancer patients was 2%, with an area under the curve (AUC) of 0.833 (*p <*0.001; **Figure 3**). Importantly, cytology samples containing >2% tumor volume could detect pathogenic mutations with a sensitivity of 69.2% (9/13).

**Table 4:** NGS results of cells obtained from ascites and peritoneal washing fluid (n = 33).

Patient	Stage	Cytology sample	Cytologic diagnosis	Tumor volume in cytology samples	Cytologic NGS results
PC1	pT2N0Mx	Ascites	Atypia	0.1%	CDH1 E880K (4.9%)
PC2	pT3NoMx	Ascites	Atypia	0.2%	Wild type
PC3	cT3NxMx	Ascites	Atypia	0.1%	Wild type
PC4	cT2N1M1	Ascites	Atypia	0.5%	Wild type
PC5	cT2N1Mx	Ascites	Suspicious for malignancy	2%	Wild type
PC6	pT4N0Mx	Ascites	Suspicious for malignancy	1%	Wild type
PC7	cT4NxMx	Ascites	Suspicious for malignancy	2%	Wild type
PC8	cT3N2M1	Ascites	Malignancy	10%	KRAS G12D (2.6%),
PC9	cT3NxM1	Ascites	Malignancy	2%	Wild type
PC10	cT2N2M1	Ascites	Malignancy	3%	KRAS G12D (6.9%),
PC11	cT3N2M1	Ascites	Malignancy	5%	KRAS G12D (3.4%), TP53 G245S (3.9%)
PC12	pT2N1Mx	Ascites	Malignancy	4%	KRAS G12D (2.7%), TP53 R273H (1.8%)
PC13	cT2NxM1	Ascites	Malignancy	5%	KRAS G12V (5.3%),
GC1	cT4N(+)Mx	Ascites	Malignancy	5%	TP53 E339K (52.2%)
GC2	pT4N3Mx	Ascites	Malignancy	7%	APC G253S (9.6%)
GC3	pT3N0Mx	Ascites	Malignancy	7%	KRAS G12R (3.4%)
GC4	pT4N3Mx	Ascites	Malignancy	8%	Wild type
GC5	cT4N0M1	Ascites	Malignancy	4%	Wild type
GC6	cT4N(+)Mx	Ascites	Malignancy	8%	KRAS G12S (16.1%)
GC7	cT4N(+)Mx	Ascites	Malignancy	9%	Wild type
GC8	cT4N(+)Mx	Ascites	Malignancy	10%	Wild type
GC_W1	pT4N3Mx	Peritoneal washing	Atypia	0.1%	Wild type
GC_W2	pT3N2Mx	Peritoneal washing	Suspicious for malignancy	0.5%	Wild type
GC_W3	pT4N3Mx	Peritoneal washing	Malignancy	1%	Wild type
GC_W4	pT1N0Mx	Peritoneal washing	Negative for malignancy	0%	Wild type
GC_W5	pT1N0Mx	Peritoneal washing	Negative for malignancy	0%	Wild type
GC_W6	pT1N0Mx	Peritoneal washing	Negative for malignancy	0%	Wild type
MES1	Not applicable	Ascites	Benign mesothelial hyperplasia	0%	Wild type
MES2	Not applicable	Ascites	Benign mesothelial hyperplasia	0%	Wild type
MES3	Not applicable	Ascites	Benign mesothelial hyperplasia	0%	Wild type
MES4	Not applicable	Ascites	Benign mesothelial hyperplasia	0%	Wild type
MES5	Not applicable	Ascites	Benign mesothelial hyperplasia	0%	Wild type
MES6	Not applicable	Ascites	Benign mesothelial hyperplasia	0%	Wild type

**Figure 4:**
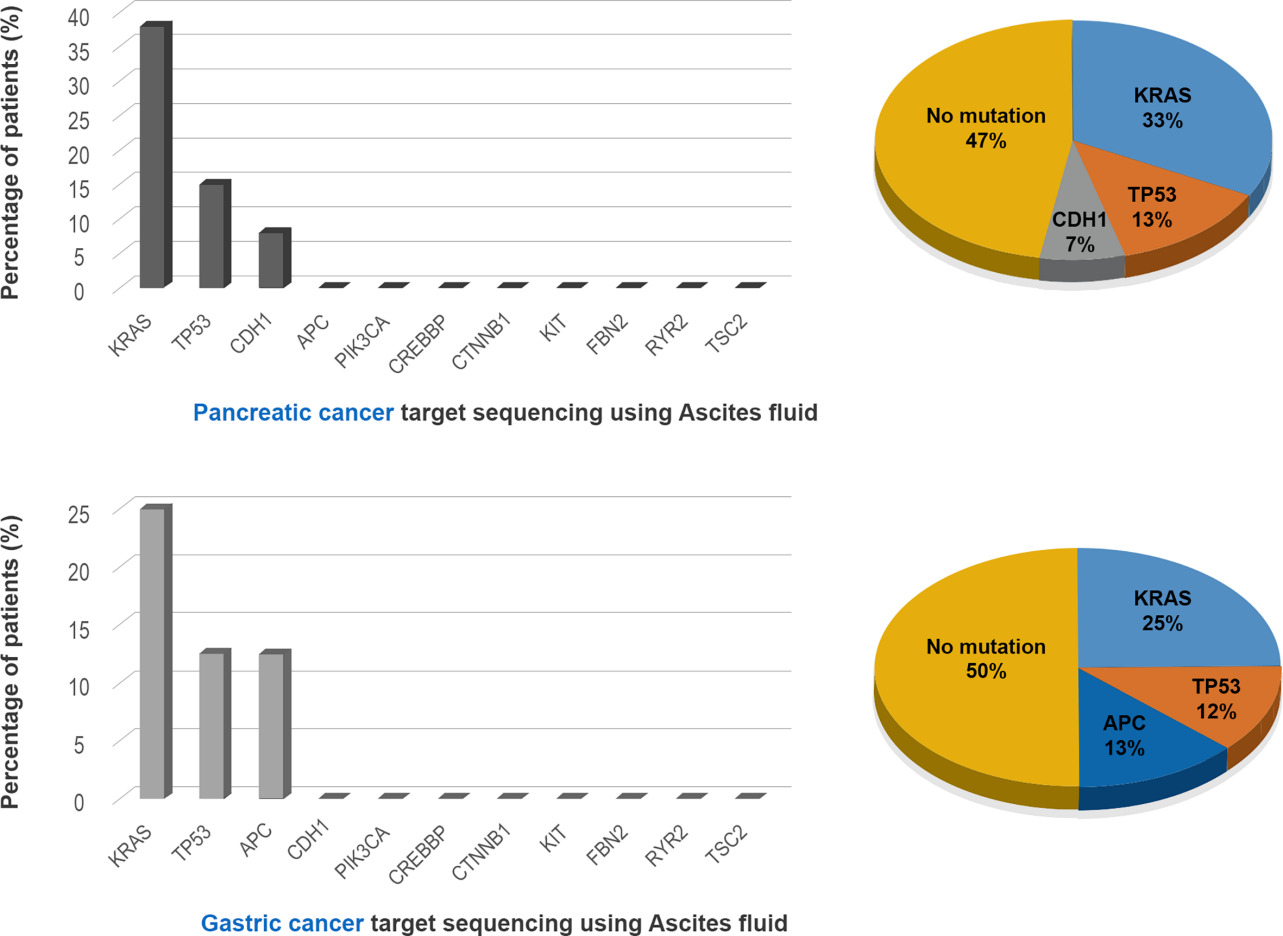
Detection of mutated genes in the ascitic fluid in the pancreatic and gastric cancer patients.

The NGS results for ascites cytology are shown in **Figure 4**. Of the ascites samples from patients with pancreatic cancer, 33, 13, and 7% were positive for KRAS, TP53, and CDH1 mutations, respectively. Similarly, 25, 12, and 13% of ascites samples from patients with gastric cancer were positive for KRAS, TP53, and APC mutations, respectively. Complete comparative results are shown in **Supplementary File 2**.

## Discussion

Targeted anti-cancer agents require that patients be genotyped, thereby selecting patients likely to respond to treatment. Genotyping using NGS can decide treatment plans and predict prognoses for cancer patients (9). NGS for genotyping applications requires a certain amount of tumor DNA. However, it may not be feasible to collect tumor samples from some patients because of tumor location, the risk of tumor spread, and complications during the biopsy or surgical procedure (10). Liquid biopsy is the sampling of non-solid biological tissue, such as blood and body fluids (e.g. urine, saliva, ascites, or effusion fluids). Liquid biopsy represents non-invasive alternative to tissue biopsy and allows a longitudinal evaluation for cancer evolution (11). Cytology samples are one of the classic and conventional liquid biopsies (12). Cytology samples can be easily obtained and repetitively sampled for alternative to tissue sampling or current status evaluation of cancer patients. DNA extracted from these cells or liquid biopsy provide genomic materials for molecular testing and for morphologic evaluation, thereby avoiding additional biopsies (13). Cytology samples can be a source for molecular genetics and diagnostics.

The present study evaluated the ability to detect genomic mutations in cell samples obtained from ascites and peritoneal washing samples of patients with GI cancers. The diagnostic value of detecting somatic mutations in these samples was determined by evaluating paired tumor tissue samples. The overall agreement between cytology and tissue genotyping results was 25%, with a sensitivity of 21.43% and a specificity of 50%. The agreement between ascites and tissue samples was 27%, whereas the agreement between peritoneal washing and tissue samples was 0%. Targeted genes with high mutational frequency in the primary tumor samples showed higher mutation detection rate in the cytology samples, with one patient (PC1) with a pathogenic CDH mutation having AFs of 4.6% in ascites and 54.1% in tumor tissue. Genotyping results were also compared with tumor volume and cytologic diagnosis in 33 patients, including 27 with GI cancers and six with non-malignant diseases. Tumor volume in the ascites/peritoneal washing samples ranged from 0 to 10%, with the optimum cutoff tumor volume to detect somatic genotype being 2%. Remarkably, ascites/peritoneal washing samples containing >2% tumor volume could detect pathogenic mutations with a sensitivity of 69.2%.

The relationship between tumor volume in the ascites/peritoneal washing samples and the detection of somatic mutations was likely affected by the large amounts of background mesothelial and inflammatory cells in these samples. Use of the detected threshold cutoff for tumor volume could improve diagnostic sensitivity. High mutation frequency in the primary tumor was associated with increased mutant DNAs in the ascites/peritoneal washing samples, allowing primary tumors with high mutational burden to be utilized for cytomolecular evaluation. Samples previously evaluated for cyto-molecular NGS testing include FFPE cell blocks, ascites/peritoneal washing supernatants, cell pellets, direct smears, cells scraped from slides, and additional aspirated samples (14–16). NGS analysis of FFPE cell blocks from two pancreatic cancer patients and supernatants of ascitic fluid from three gastric and one colon cancer patients showed concordant results with tissue samples (14, 17). Other studies evaluated cell free DNA (cfNDA) from ascitic fluid and detected TP53, EGFR, ALK, BRAF from ovary and lung cancer patients using duplex sequencing or PNA-Q-PCR (18–20).

To our knowledge, no previous study to date had evaluated the results of NGS-based analysis of cells obtained from peritoneal washing fluid. In the present study, NGS analysis of all peritoneal washing cytology showed wild type, regardless of tumor stage or tumor volume, suggesting that evaluation of genomic mutations in these samples was limited by the very low tumor volume. By contrast, pleural effusion samples obtained from lung cancer patients have provided good diagnostic and prognostic information (21). These findings suggest that the types of cancer, types of sample, tumor volume in the sample, and the mutational status of the primary sample could affect cytomolecular results.

Cytologic diagnosis frequently includes ambiguous results, such as atypia. Undetermined cytology may therefore require repetitive sampling, and clinical and radiologic consensus. NGS analysis may help in the diagnosis of patients with ambiguous results, such as atypia. A somatic mutation was detected in the ascitic fluid, containing 0.1% tumor volume, of one patient (PC1) previously diagnosed with atypia. Cytomorphologic diagnosis depends on the volume of tumor cells in the analyzed specimen. Patients with very low tumor volume (~0.1–0.5%) showed atypia, and those with low tumor volume (~1–2%) were found to be suspicious for malignancy. Tumor volume in the cytology samples also affected the detection of somatic mutations in patients with GI cancers. Therefore, NGS is limited in improving the diagnosis of ascites/peritoneal washing samples. However, NGS-based genotyping can help reduce false positive results because wild-type genotypes were detected in all ascites samples from patients with non-malignant conditions, thus ruling out malignancy.

NGS results obtained from ascites of patients with pancreatic cancer showed that 33, 13, and 7% were positive for KRAS, TP53, and CDH1 mutations, respectively, whereas analysis of ascites samples from gastric cancer patients showed that 25, 12, and 13% were positive for KRAS, TP53, and APC mutations, respectively. Because similar genetic mutations are present in gastrointestinal and hepatobiliary tract cancers, patients with pancreatic and gastric cancers did not show significant molecular differences. Cytology-based NGS results cannot determine the origin of tumor cells in the GI tract but may reflect tumor dynamics. Interestingly, a novel KRAS mutation, not present in the primary tumor sample, was detected in the ascitic fluid of one gastric cancer patient (GC3). Cytology-based NGS testing can reflex current mutational status and genomic resistance that may help predict and manage clinical disease.

The present study had several limitations. Due to the small number of patients, the results provide less definitive conclusions regarding the effectiveness of NGS based targeted sequencing using ascites and peritoneal washing of GI cancer patients. In our retrospective study, frozen stored cytology samples could affect mutational output due to archiving status. In addition, ascites commonly occurred as the evidence of recurrence during the follow-up after the surgery. The time interval between collected paired cytology and tissue samples were 0–42 months in our study and that could explain discrepant results of compared samples. Ascites and peritoneal washing commonly have very low cellularity due to reactive mesothelial cells lining the abdominal cavity, and most collected samples had less than 2% tumor cells of the whole volume (22). False negative results limited the ability to identify mutational status and help to predict recurrence. NGS based targeted sequencing of cytology could give additional supportive information for the interpretation of GIcancer patient status, but must be considered along with other clinical and radiologic findings.

To our knowledge, this study is the first to evaluate molecular aberrations in ascites and peritoneal washing fluid in patients with GI cancers. These molecular aberrations can predict tumor recurrence and patient prognosis, and may help in determining treatment. Various liquid biopsy samples, including effusion, exfoliated, and washing samples, and various preparations, including cell pellets, cell supernatants, and cell blocks, can be utilized for cytomolecular evaluation of patients with GI cancers. Further studies involving larger numbers of ascites patients and a prospective design are required to demonstrate the clinical validity of targeted sequencing of ascites cytology samples.

## Data Availability Statement

The datasets presented in this study can be found in online repositories. The names of the repository/repositories and accession number(s) can be found in the article/**Supplementary Material**.

## Ethics Statement

The studies involving human participants were reviewed and approved by IRB file no. CNUH 2020-09-015. Written informed consent for participation was not required for this study in accordance with the national legislation and the institutional requirements.

## Author Contributions

Resources and data curation; S-HK. Conceptualization and writing—review and editing; M-KY. Conceptualization and validation; J-MK and M-KY. Formal analysis, M-KC. Supervision, writing-original draft, and writing—review and editing; GB and M-KY. All authors contributed to the article and approved the submitted version.

## Funding

This study was supported by the grants from the Basic Science Research Program through the National Research Foundation of Korea (NRF) funded by the Ministry of Education (2017R1D1A1B04031187), the Bio and Medical Technology Development Program of the National Research Foundation (NRF) funded by the Korean government (MSIT) (2019M3E5D1A02068558), and Korea Health Technology R&D Project through the Korea Health Industry Development Institute (KHIDI), funded by the Ministry of Health & Welfare, Republic of Korea (grant number: HR20C0025).

## Conflict of Interest

The authors declare that the research was conducted in the absence of any commercial or financial relationships that could be construed as a potential conflict of interest.

## Supplementary Material

Supplementary File 1 Sequencing data of all samples.

Supplementary File 2 Information for comparison results.
